# Alignment of Research Efforts With the Diabetic Retinopathy Burden of Disease and Socioeconomic Factors: An Analytical Bibliometric Study

**DOI:** 10.34172/ijhpm.9345

**Published:** 2026-04-11

**Authors:** Farbod Semnani, Seyed Sahab Aarabi, Kiana Hassanpour, Payam Kabiri, Mojtaba Sedaghat, Amirhossein Takian

**Affiliations:** ^1^National Center for Health Insurance Research, Tehran, Iran.; ^2^School of Medicine, Tehran University of Medical Sciences (TUMS), Tehran, Iran.; ^3^Ophthalmic Research Center, Research Institute for Ophthalmology and Vision Science, Shahid Beheshti University of Medical Sciences, Tehran, Iran.; ^4^Department of Biostatistics and Epidemiology, School of Public Health, Tehran University of Medical Sciences (TUMS), Tehran, Iran.; ^5^Department of Community Medicine, Faculty of Medicine, Tehran University of Medical Sciences, Tehran, Iran.; ^6^Department of Global Health and Public Policy, School of Public Health, Tehran University of Medical Sciences (TUMS), Tehran, Iran.; ^7^Department of Health Management, Policy and Economics, School of Public Health, Tehran University of Medical Sciences (TUMS), Tehran, Iran.; ^8^Health Equity Research Centre (HERC), Tehran University of Medical Sciences (TUMS), Tehran, Iran.

**Keywords:** Diabetic Retinopathy, Disease Burden, Bibliometric Analysis, Research Inequity, Socioeconomic Factors, Global Health

## Abstract

**Background:**

Diabetic retinopathy (DR) is a major microvascular complication of diabetes. Given its growing global burden and research inequities, we examined how national DR burden and socioeconomic factors relate to research interest (RI) in DR across low- and middle-income countries (LMICs) and high-income countries (HICs).

**Methods:**

We retrieved peer-reviewed DR articles published between 2018 and 2022 from Scopus. Spearman’s correlation test and multivariable linear regression were used to assess the associations between the independent variables: (*a*) socioeconomic factors, including health expenditure per capita purchasing power parity (PPP), current health expenditure (% of gross domestic product [GDP]), research and development (R&D) index, and human development index (HDI); (*b*) age-standardized years lived with disability (YLDs) rates of DR-related moderate (moderate vision impairment, MVI) and severe vision impairment (SVI) and blindness; and (*c*) disability-adjusted life years (DALYs) rate for diabetes, with our outcome variable, DR RI, calculated as the ratio of the number of DR publications in the field of medicine and life sciences to the whole output in the same field and country. Sensitivity analyses (2020-2022 RI vs. 2018-2019 burden) were conducted to address temporality.

**Results:**

In HICs, after adjustment for socioeconomic factors and DR-specific burden (MVI, SVI, or blindness, modeled separately), the diabetes DALY rate was the only variable independently associated with RI: MVI (β=0.56, 95% CI: 0.16 to 0.95), SVI (β=0.57, 95% CI: 0.19 to 0.94), and blindness (β=0.61, 95% CI: 0.21 to 1.02). In LMICs, no significant relationships were found between RI and MVI, SVI, blindness or diabetes DALY rates. These findings remained consistent in sensitivity analyses.

**Conclusion:**

Our research demonstrates that there is a lack of significant correlation between research efforts and the burden of DR, particularly among LMICs, which may highlight the need to strengthen research infrastructure and realign national health research priorities.

## Background

**Key Messages**

**Implications for policy makers**

- Establish targeted funding: National and international funding agencies must establish targeted funding calls that will bridge the gap in diabetic retinopathy (DR) research in low- and middle-income countries (LMICs), focusing on implementation science and health system investigations.
- Facilitating high-income country (HIC)-LMIC collaboration: Policy-makers in the HICs should incentivize and facilitate equitable research collaboration based on the local priorities, data, and capacity development of the LMIC partners, rather than the HIC-dominated research orientations.
- Enhance national research infrastructure: The national governments of LMICs can be assisted in the creation of local research infrastructure, which can shift the focus from general global trends to covering the gap of the local disease burden, such as the growing incidence of DR and diabetes.
- Integrate research assessment with health endpoints: Health ministries must also put the appropriate metrics, including the ratio of the national disease burden (years lived with disability, YLDs) to the product of research, in their publicly funded research assessment systems.

**Implications for the public**

Diabetic retinopathy (DR), a blinding eye disease linked to diabetes, is a leading cause of preventable blindness, particularly among working-age adults. Our research emphasizes a concerning gap: in many of the countries most affected by this disease, very little is being done to improve screening, treatment, and prevention methods. This is especially true in low- and middle-income nations. For the public, this means that the health issues that matter most to you might not be receiving the scientific focus they deserve. Our findings serve as a call to action for governments and funding agencies around the world to shift research efforts toward the health challenges that are most pressing for local communities. This ensures that everyone, everywhere, can benefit from medical advancements and have a better chance of preserving their vision.

Diabetic retinopathy (DR) is a severe and common microvascular complication of diabetes mellitus.^1^ The International Diabetes Federation estimated the global diabetic population to be 589 million people in 2024 (11.1% of adults) and predicted that this index would reach 853 million by 2050.^2^ On average, almost one-third of diabetic patients have some form of DR. Among people with DR, one-third have vision-threatening DR.^3^ This corresponds to 5% to 10% of people with diabetes overall.^4^ DR is the leading cause of preventable blindness in working-aged people^5^ and the fifth greatest contributor to moderate to severe vision impairment (MSVI) and blindness in adults aged 50 and older. The other four leading causes of MSVI and blindness in the Global Burden of Disease (GBD) estimates in 2020 include under-corrected refractive error, cataract, age-related macular degeneration, and glaucoma. Notably, unlike these other causes, the age-standardized prevalence of all these causes decreased from 1990 to 2020.^6^ In particular, the age-standardized prevalence of DR increased from 14.9% to 18.5% from 1990 to 2020, with the largest increase occurring in southern sub-Saharan Africa (138.3%).^6^ The demographic changes and rapid aging of the global population, the increased life expectancy of the general population, ie, people with diabetes, and lifestyle changes underlying the increased risk of developing diabetes may explain this phenomenon and predict a rocketing demand for care and eye treatment in the coming years, representing a major public health challenge worldwide.^7^

 In 2019, approximately 80% of people with diabetes lived in low- and middle-income countries (LMICs).^8^ Previous studies indicated a greater incidence of DR in western high-income countries (HICs) than in Asian LMICs (the USA and Canada vs. China, India, and South Korea).^9^ However, recent studies suggest that the actual prevalence of DR in Asian countries could be greater than expected. Indeed, the lower rates of reported DR in LMICs might be due to a lack of awareness, late diagnosis of DR, and poor access to healthcare in such settings.^7^ While the incidence of blindness associated with DR is decreasing in HICs, probably due to increased awareness, timely screening, and appropriate treatment, LMICs are experiencing an increasing trend of blindness.^10^ On the other hand, while the population of adults with diabetes in LMICs is expected to increase by 69% from 2010 to 2030, the index is projected at only 20% for HICs.^11^

 Previous bibliometric analyses have primarily described the publication trends in DR^12-17^; however, the relationship between research output and disease burden has not been quantitatively explored. Considering the rising burden of DR, existing inequities in biomedical research, incongruent attention to research topics related to local needs (eg, burden of disease) within countries,^18^ and the scarcity of relevant published data, this study aims to outline the trends of DR research conducted in different countries (categorized as HICs and LMICs). Our work aims to shed light on the level of correlation between DR research interest (RI) and the national burden of the disease and socioeconomic factors. Using a novel analytic framework to quantify this association, the present study could introduce new insights into global research inequalities and priority setting.

## Methods

###  Study Design

 This is a cross-sectional analytical bibliometric study using national-level data.

###  Database

 We used the Scopus database to extract the bibliometric data. Scopus is the largest and most comprehensive available scientific database,^19^ encompassing nearly all studies indexed in PubMed and providing journal coverage twice as large as that of the Web of Science.^20,21^ Additionally, Scopus is more representative of the true contribution of each country to the literature due to its broader publication landscape and journals’ impact factors. It is more inclusive of journals with lower impact factors, which are more accessible to LMICs.^22^ Moreover, Scopus is a comprehensive database that provides citations from diverse disciplines, including health, social, physical, and life sciences, and is highly suitable for multidisciplinary DR research.^23,24^

###  Search Strategies

**Figure 1:**
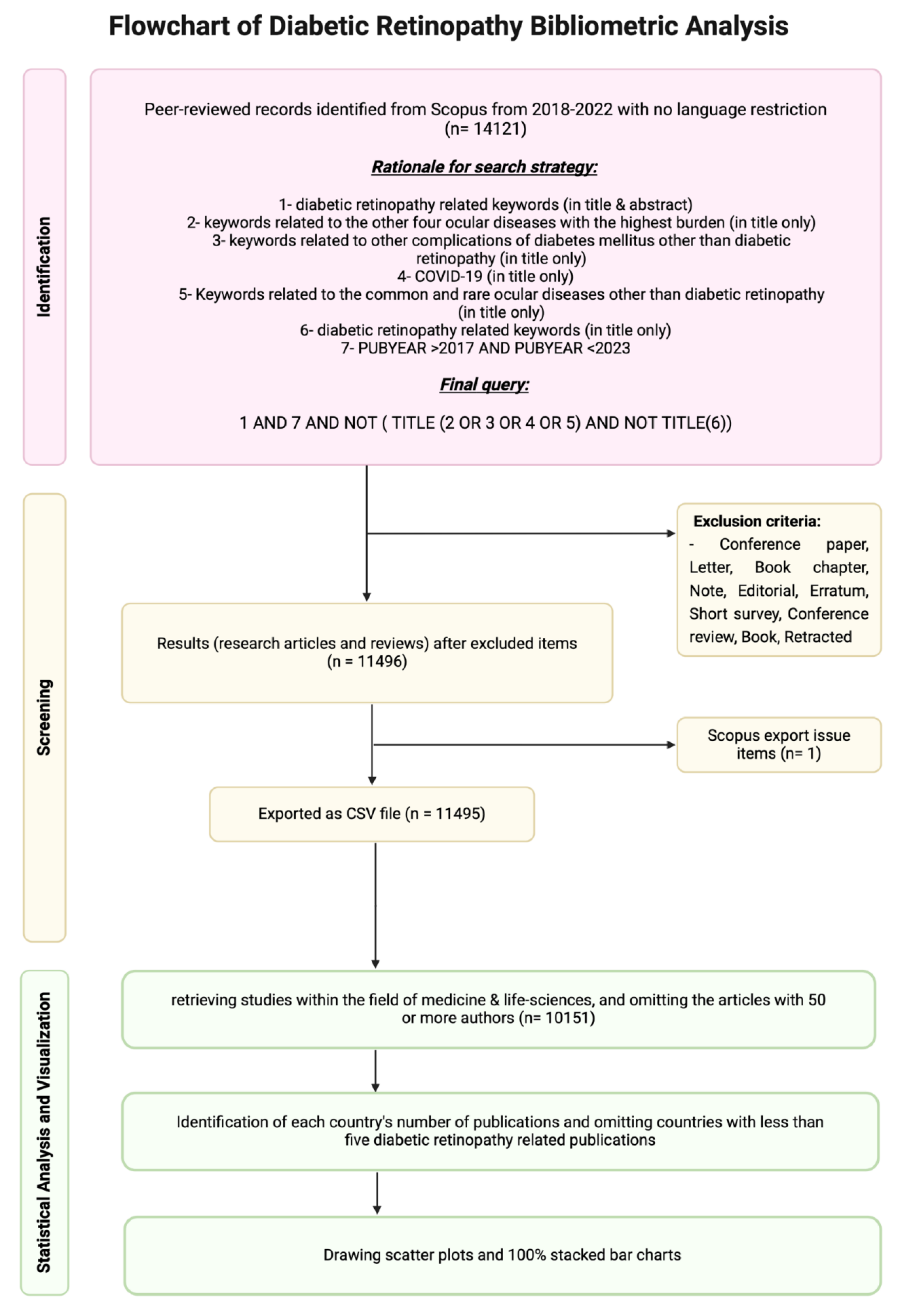
The Flowchart of the Search Strategy, Inclusion and Exclusion Criteria and the Number of Studies Retrieved in Each Stage.

 We developed a comprehensive search strategy for a 5-year interval between 2018 and 2022 and validated it through multiple experiments with different scenarios to achieve minimum false negative and false positive results (Figure 1). Previous studies have mentioned that the title/abstract search strategy may retrieve too many false-positive and false-negative results.^25,26^ It is also known that the title search will not be comprehensive,^27^ particularly for assessing country-specific contributions to this field. Thus, we used a validated title/abstract search strategy.

 To increase search efficiency, we developed a composite search strategy using Boolean operators, truncation symbols, and the proximity operator of Scopus (W) from the keywords related to DR, using insights from Medical Subject Headings (MeSH) of PubMed and some previous related systematic reviews.^20,21,28^ In addition, we automatically excluded the articles not investigating DR per se, particularly those four ocular diseases with the highest burden (ie, under-corrected refractive error, cataract, age-related macular degeneration, and glaucoma), COVID-19, and all other common and rare ocular diseases, by incorporating their relevant keywords in our query. Our approach was to exclude results with specific keywords related to these diseases irrelevant to DR in the title, only if DR-related keywords were absent in the title and present only in the abstract. Furthermore, to enhance credibility, two authors (KH, an ophthalmologist, and PK, a bibliometric expert) reviewed and approved the search strategies. We followed the same path to retrieve related articles on the other four ocular diseases with the highest burden, namely, under-corrected refractive error, cataract, age-related macular degeneration, and glaucoma. The detailed search queries can be found in Supplementary file 1.

 Like many previous bibliometric studies, we included all peer-reviewed published studies and excluded conference papers, notes, editorials, erratas, letters, and book chapters.^25,29,30^ As all records in Scopus have English abstracts, no language restriction was implemented. Subsequently, we deleted the retracted articles which were automatically marked by SciVal. We performed and finalized the Scopus search on February 4, 2024 to prevent minor daily updates of Scopus that could potentially alter the final results.

####  Validation

 The authors followed a validation process adapted from previously published bibliometric studies.^25,27,30^ Accordingly, to rule out the presence of false-positive results, two authors (FS and SSA) independently reviewed the top 100 highly cited results for irrelevant records, resolved the conflicts by further discussion and senior author (AT) mediation, and revised the search syntax until no false-positive articles could be detected. The validity of the minimum number of false-negative (missing) records was confirmed by comparing the data obtained for the 10 most productive authors with the data present in their Scopus personal profiles.^27^ For example, our search query retrieved these publications for the following authors: Wong, T.Y. (n = 90 documents), Sivaprasad, S. (n = 85 documents), and Bandello, F. (n = 65 documents), while their Scopus profiles contained 93, 86, and 67 DR-oriented true outputs, respectively. The interclass Spearman correlation coefficient was 0.997, and the *P* value was <.001, suggesting the high validity of our search strategy (Table S1, Supplementary file 2).

###  Variables and Outcomes

####  Bibliometric Variables (Dependent Variables)

 The primary variables of interestof the present study include the crude number of DR publications and the DR RI at a national level. After conducting the search process, the specific country contribution numbers were calculated based on the country affiliation for each author on an article, regardless of his/her position. For instance, if an article was published by two authors, one from Iran and one from Turkey, then the article was counted once for Iran and once for Turkey. An article with authors with the same country affiliation was counted only once for that country. This type of labeling is an automatic practice performed by Scopus and SciVal (a bibliometric analytics platform that utilizes Scopus data to evaluate research performance).^27,31^

 To calculate the DR RI, the number of DR-oriented publications of each country in the field of medicine & life sciences was divided (adjusted) by the total number of publications in the same field in the same reference period, 2018-2022. For instance, if country A has published 100 DR papers out of 50 000 total medical publications (RI = 0.002), while country B has published 50 DR papers out of 10 000 total (RI = 0.005), country B has a higher RI despite lower absolute DR output. We chose the total number of national publications in Medicine & Life Sciences as our denominator because it best aligns with the health-related focus of DR and is most compatible with each country’s real biomedical research output.

 In addition, citation comparison was performed using the field-weighted citation impact, a proxy indicator of the scientific impact of publications. This metric compares the number of citations received by an entity’s publications with the mean number of citations received by all other similar publications (the same publication year, publication type, and discipline according to Scopus subject classification systems) in the whole literature.^32^

 To assess the relationship between disease burden and the RI, we restricted the results to the items published between 2018 and 2022, which are included within publications recognized under the “Medicine & Life Sciences” in the Quacquarelli Symonds Classification. This consists of five subjects each mapped to one or more subjects of the All-Science Journal Classification, the default of Scopus for categorizing publications into scientific disciplines. In particular, it encompasses all publications indexed in at least one health or life science-related field (ie, dentistry, health professions, medicine, and/or nursing).^22^ It is critical to note that the subject assignment is not exclusive, and a single article might have been listed in both the medical and engineering subcategories. In addition, we excluded articles with 50 or more authors because they may not reflect the true contribution of the countries corresponding to the affiliated authors to the research of DR. This threshold was selected based on expert bibliometric consultation to ensure that our metrics reflect genuine national-level priorities rather than global consortium-driven agendas.

####  Burden of Disease and Socioeconomic Factors (Independent Variables)

 To assess the burden of DR, the cause-specific age-standardized YLDs (years lived with disability) rates of moderate vision impairment (MVI), severe vision impairment (SVI), and blindness attributed to DR were obtained from the Institute for Health Metrics and Evaluation results tool.^33^ We followed the same process for refractive diseases, age-related macular degeneration, and glaucoma, as GBD level 4 causes of death and injury. Moreover, we used the same database to extract the diabetes burden as age-standardized disability-adjusted life year (DALY) rates at the national level. The diabetes DALY rate was included to control for the confounding effect of the broader systemic health focus on diabetes mellitus. Although DR is a complication of diabetes, and adjusting for diabetes burden may lead to over-adjustment bias, its inclusion was necessary to determine if RI is driven specifically by visual impairment or more generally by the parent disease burden. Specifically, we selected and extracted data regarding “Diabetes Mellitus” from the GBD level 3 causes of death and injury and simultaneously MVI, SVI, and blindness from GBD estimates of impairment. All burden data were averaged for 2018 and 2019 and extracted for all countries and territories.

 We classified countries into LMIC or HIC groups according to the World Bank’s yearly classifications for each country.^34^ The final tag was determined based on the most common category of a country within the study’s five-year period (2018-2022) to ensure that temporary fluctuations in income did not bias the stratified analysis.^22^ Moreover, we gathered the metadata regarding the health expenditure per capita purchasing power parity (PPP) in US dollars, current health expenditure (% of gross domestic product [GDP] allocation), research and development (R&D) index, and human development index (HDI), as of the study mid-year (2020) from the World Bank.^35^ Where inconsistencies or missing values were identified, those countries were excluded from the analytical analyses but retained in descriptive summaries.

###  Statistical Analysis

####  Correlation Analysis

 We performed Spearman rank correlation analysis (certain variables not normally distributed) to identify independent variables associated with the main outcome, the RI, for DR research.^36^ The interpretation of ρ was performed according this rule of thumb: a ρ below 0.16 was considered negligible, values between 0.16 and 0.29 indicated a weak to low relationship, values between 0.3 and 0.49 showed a moderate to low correlation, values between 0.5 and 0.69 represented a moderate correlation, values between 0.7 to 0.89 represented a strong relationship and values between 0.9 and 1 were classified as very strong correlations.^37^

 The three age-standardized YLD metrics (MVI, SVI, and blindness attributable to DR) were analyzed separately to assess the consistency of associations across different severity levels of disease burden. These indicators represent overlapping components of the overall DR burden rather than independent constructs, and were therefore interpreted in a complementary manner to evaluate whether research attention aligns with varying degrees of visual impairment severity. Due to the exploratory nature of this study and the number of correlations tested, we prioritized the interpretation of effect sizes and consistent trends across models over marginal *P* values to minimize the risk of type I error.

####  Regression Analysis

 We constructed separate multivariable linear regression models for HICs and LMICs to examine whether the relationships between DR burden (MVI, SVI, and Blindness) and national RI persisted after adjustment for socioeconomic factors and overall diabetes burden. The dependent variable in all models was the RI for DR. Independent variables included the age-standardized YLD rates for MVI, SVI, and blindness due to DR (averaged for 2018–2019).

 To overcome multicollinearity and to keep variance inflation factors (VIFs) below 3,^38^ the HDI and health expenditure per capita (PPP) were selected as the primary socioeconomic covariates from among four initially tested indicators (HDI, health expenditure per capita, current health expenditure as % of GDP, and R&D index) because these variables were highly correlated.^38^ The diabetes DALY rate was also included as an additional confounder, reflecting the broader systemic burden of diabetes that may influence both DR burden and research output.

 The DR RI was right-skewed and thus normalized, transformed and presented as natural logarithms.^29^ Model assumptions were verified through residual diagnostics, normal probability plots, and collinearity assessment (all VIFs < 3). Model fit was evaluated using the adjusted R^2^.

####  Sensitivity Analysis

 To partially address temporal overlap between disease burden (2018–2019) and publication period (2018–2022), a sensitivity analysis was performed using RI values restricted to 2020–2022, with the same 2018–2019 burden and covariates. This lagged approach assessed whether prior disease burden is associated with subsequent research activity.

 We used STATA version 17.0 (StataCorp. 2021. *Stata Statistical Software: Release 17*. College Station, TX: StataCorp LLC.) and R version 4.3.1. for analyses. A two-sided *P* < .05 was used as the criterion for statistical significance.

###  Visualization

 The adjusted number of DR publications and the YLD rates for MVI, SVI, and blindness were right-skewed. We therefore applied natural logarithmic transformation to these variables for normalization and visualization.^18^ We constructed scatter plots with two-by-two grid lines overlaid on the median values of each axis (quadrant plots).^39^ These visualizations categorized countries in each income group according to the relationship between DR burden and RI. These are useful plots for the depiction of quantitative datasets, especially census data, which do not necessarily show a pattern, but every point on the map is note-worthy.^40^

 In particular, these quadrant plots were utilized to classify each country into one of the following four categories, as represented earlier by a study in the Scientometrics journal^41^: “Low relative RI, Low relative disease burden,” “Low relative RI, High relative disease burden,” “High relative RI, Low relative disease burden,” or “High relative RI, High relative disease burden.” The final label of each country was decided upon its most common category out of the three discrete tags corresponding to the three exclusive values of the DR burden.

 Subsequently, we performed the same categorization and labeling process for each country in the LMICs and HICs subgroups separately, based on the concordance of the relative DR burden (age-standardized YLDs of MVI, SVI, and blindness) with the relative publication output among the five ocular diseases with the highest burden. This was achieved by drawing proportional stacked (100%) bar charts, which are conveniently used for *relative* comparisons of individual attributes.^42^ Four stacked bar charts were drawn for LMICs and HICs, the first representing the ascendingly sorted relative publication output and the second to fourth representing the relative DR burden (age-standardized YLDs of MVI, SVI, and blindness) among these five diseases. The concordance was translated into the four previously mentioned categories, using medians of these proportional values as thresholds and with the final label decided on the consensus of the three discrete tags of the DR burden.

## Results

 After the exclusion of non-peer-reviewed records, the final dataset consisted of 9832 research articles and 1663 reviews, translating into a total of 11 495 eligible documents. After exclusion of 17 articles with >50 authors, a total of 10 151 DR-related articles within medicine and life sciences field were included in our final analysis.

###  Global Research Landscape and Distribution

 The overall research output on DR demonstrated a consistent upward trend during the study period (Figure S1, Supplementary file 3). The most productive country was China, which contributed 2932 documents (28.9% of the total), followed by the United States with 1964 documents (19.3%). A cartogram illustrates that global research output is concentrated in North America, Western Europe, and East Asia, while regions with a significant diabetes burden, such as Africa and South America, have markedly lower research productivity (Figure S2, Supplementary file 3). This geographical distribution of research output contrasts with the global burden of DR, which is most severe in regions including the Middle East, Sub-Saharan Africa, and Latin America (Figure S3, Supplementary file 3).

###  Comparative Analysis of HICs and LMICs

 Our final dataset comprised 44 HICs and 44 LMICs. The RI was comparable between LMICs (0.00148) and HICs (0.00146) (*P =*.54). However, a significant disparity was observed in scientific impact; the mean field-weighted citation impact for publications from HICs was 1.65 (SD = 0.88), significantly higher than the mean of 1.22 (SD = 1.38) for LMICs (*P *<.001).

 The average RI for glaucoma was similar between LMICs (0.0017) and HICs (0.0018) (*P* = .55), as well as for cataract (0.0016 vs. 0.0013; *P* = .13) and refractive disorders (0.0011 vs. 0.0010; *P* = .99). In contrast, the RI for age-related macular degeneration was significantly higher in HICs compared with LMICs (0.0010 vs. 0.0005; *P* < .001).

###  Correlation of Research Output With Disease Burden and Socioeconomic Factors

 Spearman’s rank correlation analysis was performed to assess the relationships between research output (both crude publication numbers and RI) and key variables.

####  Correlation With Crude Publication Numbers

 As detailed in Table 1, the crude number of DR publications in HICs showed a moderate positive correlation with health expenditure per capita (PPP) (ρ = 0.55, *P*< .001), current health expenditure as a percentage of GDP (ρ = 0.58, *P*< .001), the R&D index (ρ = 0.57, *P*< .001), and a moderate to low positive correlation with the HDI (ρ = 0.46, *P*< .01). In contrast, among LMICs, only the R&D index demonstrated a significant moderate positive correlation with the number of publications (ρ = 0.50, *P*< .01). The age-standardized YLD rate for MVI was negatively correlated with publication volume in HICs (ρ = -0.34, *P =*.03), while the YLD rate for blindness showed a moderate to low positive correlation in LMICs (ρ = 0.32, *P =*.05).

**Table 1:** Spearman’s Rank Correlation Coefficient (Rho) of Crude Number of DR Publications With Age-Standardized DR-Attributable YLD Rates of MVI, SVI, and Blindness, and With Health Expenditure Per Capita PPP, Current Health Expenditure (% of GDP), R&D, and HDI

	**MVI**		**SVI**		**Blindness**		**Health Expenditure Per Capita (PPP)**		**Current Health Expenditure (% of GDP)**		**R&D**		**HDI**	
**Category**	**LMIC**	**HIC**	**LMIC**	**HIC**	**LMIC**	**HIC**	**LMIC**	**HIC**	**LMIC**	**HIC**	**LMIC**	**HIC**	**LMIC**	**HIC**
Rho (ρ)	0.15	-0.34	0.19	-0.06	0.32	0.01	0.17	0.55	0.02	0.58	0.50	0.57	0.14	0.46
*P* value	.36	.03	.25	.70	.05	.98	.31	<.001	0.92	<.001	<.01	<.001	.42	<.01

Abbreviations: DR, diabetic retinopathy; MVI, moderate vision impairment; SVI, severe vision impairment; PPP, purchasing power parity; GDP, gross domestic product; R&D, research and development; HDI, human development index; YLD, years lived with disability; HIC, high-income country; LMIC, low- and middle-income country.

####  Correlation With Research Interest 

#####  a. Spearman (Bivariate) Correlation Analysis

 The analysis of RI revealed a different and more complex set of associations (Table 2). In HICs, RI had a moderate positive correlation with the national disease burden for MVI (ρ = 0.50, *P*< .001) and moderate to low correlation for SVI (ρ = 0.40, *P* = .01), but no correlation with blindness (ρ = 0.16, *P* = .30). Additionally, a positive Spearman correlation was observed between diabetes DALY rate and RI in HICs (ρ = 0.51, *P*< .001). Conversely, RI demonstrated a significant negative correlation with all measured socioeconomic factors: health expenditure per capita (ρ = -0.48, *P* = .001), current health expenditure (% of GDP) (ρ = -0.65, *P*< .001), R&D index (ρ = -0.35, *P* = .03), and HDI (ρ = -0.33, *P* = .03). In LMICs, no statistically significant correlation was found between RI and any of the DR burden metrics, socioeconomic variables or diabetes DALY rate.^30^

**Table 2:** Spearman’s Rank Correlation Coefficient (Rho) of the RI toward DR Research With Age-Standardized DR-Attributable YLD Rates of MVI, SVI, and Blindness, and With Health Expenditure Per Capita PPP, Current Health Expenditure (% of GDP), R&D, and HDI

	**MVI**		**SVI**		**Blindness**		**Health Expenditure Per Capita (PPP)**		**Current Health Expenditure (% of GDP)**		**R&D**		**HDI**	
**Category**	**LMIC**	**HIC**	**LMIC**	**HIC**	**LMIC**	**HIC**	**LMIC**	**HIC**	**LMIC**	**HIC**	**LMIC**	**HIC**	**LMIC**	**HIC**
Rho (ρ)	0.12	0.50	0.07	0.40	-0.01	0.16	- 0.05	-0.48	- 0.12	-0.65	- 0.11	-0.35	0.09	-0.33
*P* value	.49	<.001	.69	.01	.96	.30	.79	.001	.47	<.001	.53	.03	.58	.03

Abbreviations: DR, diabetic retinopathy; MVI, moderate vision impairment; SVI, severe vision impairment; PPP, purchasing power parity; GDP, gross domestic product; R&D, research and development; HDI, human development index; RI, research interest; YLD, years lived with disability; HIC, high-income country; LMIC, low- and middle-income country.

#####  b. Multivariable Linear Regression Analysis

 After adjustment for HDI, health expenditure per capita (PPP), and diabetes DALY rate, results diverged markedly by income group (Table 3).

**Table 3:** Multivariable Linear Regression Analysis of the Association Between Diabetic Retinopathy Burden (MVI, SVI, Blindness) and National Research Interest in Diabetic Retinopathy, Stratified by Country Income Level^a^

**Category**	**DR burden Model**	**Independent Variable**	**β Coefficient**	**95% CI**	***P*** ** Value**	**Adjusted R**^2^
HIC	MVI	MVI	0.05	-0.33 to 0.42	.80	0.30
		DB	0.56	0.16 to 0.95	.007	
		HDI	0.29	-0.16 to 0.74	.20	
		HEpc (PPP)	-0.33	-0.74 to 0.08	.11	
	SVI	SVI	0.03	-0.31 to 0.38	.85	0.30
		DB	0.57	0.19 to 0.94	.004	
		HDI	0.29	-0.16 to 0.73	.20	
		HEpc (PPP)	-0.33	-0.74 to 0.08	.11	
	Blindness	Blindness	-0.04	-0.38 to 0.30	.82	0.30
		DB	0.61	0.21 to 1.02	.004	
		HDI	0.29	-0.16 to 0.75	.20	
		HEpc (PPP)	-0.34	-0.75 to 0.07	.10	
LMIC	MVI	MVI	0.18	-0.22 to 0.59	.37	-0.06
		DB	-0.03	-0.45 to 0.40	.89	
		HDI	0.17	-0.39 to 0.72	.54	
		HEpc (PPP)	-0.20	-0.77 to 0.36	.47	
	SVI	SVI	0.21	-0.20 to 0.63	.31	-0.05
		DB	-0.05	-0.47 to 0.38	.82	
		HDI	0.18	-0.36 to 0.73	.50	
		HEpc (PPP)	-0.19	-0.76 to 0.37	.49	
	Blindness	Blindness	-0.13	-0.54 to 0.29	.55	-0.07
		DB	0.16	-0.28 to 0.60	.46	
		HDI	0.19	-0.37 to 0.74	.50	
		HEpc (PPP)	-0.17	-0.76 to 0.41	.56	

Abbreviations: MVI, moderate vision impairment; SVI, severe vision impairment; DB, diabetes burden; HDI, human development index; HEpc (PPP), health expenditure per capita, purchasing power parity ($); HIC, high-income country; LMIC, low- and middle-income country; DR, diabetic retinopathy; CI, confidence interval. ^a^Covariates included the HDI, health expenditure per capita (PPP, international dollars), and diabetes burden (age-standardized DALY rate).

 When HDI, health expenditure per capita (PPP) and diabetes DALY rate were included in the models, the previously observed bivariate associations between DR-attributable YLDs (MVI and SVI) and RI lost statistical significance. Instead, diabetes DALY rate emerged as the sole statistically significant variable associated with RI in all three models (MVI-model: β = 0.56, 95% CI 0.16 to 0.95, *P* = .007; SVI-model: β = 0.57, 95% CI 0.19 to 0.94, *P* = .004; Blindness-model: β = 0.61, 95% CI 0.21 to 1.02, *P* = .004). The DR YLD variables (MVI, SVI, blindness) were not significant variables in adjusted HIC models. HDI and health expenditure per capita were not statistically associated with RI in adjusted models. Adjusted R^2^ for HIC models was 0.30, indicating the model explained ~30% of variance in RI.

 In LMICs, adding HDI, health expenditure per capita (PPP), and diabetes DALY rate to the models did not change results. There were no statistically significant associations between RI and DR YLDs (MVI, SVI, blindness), diabetes DALY rate, HDI, or health expenditure per capita (PPP). Example point estimates for diabetes DALY rate in LMIC models were small and non-significant (MVI-model: β = −0.03, 95% CI −0.45 to 0.40, *P* = .89; SVI-model: β = −0.05, 95% CI −0.47 to 0.38, *P* = .82; Blindness-model: β = 0.16, 95% CI −0.28 to 0.60, *P* = .46). Model fit was poor (adjusted R^2^ = 0.00–0.02), indicating these covariates explain negligible variance in RI among LMICs.

 All independent variables had VIFs < 3 (Table S2, Supplementary file 2), indicating multicollinearity was not a concern. Residuals and normality plots did not reveal substantive violations of regression assumptions.

#####  c. Sensitivity Analysis

 To reduce concerns about simultaneity bias, we re-ran the Spearman correlations and multivariable regressions with RI restricted to 2020–2022 and the same 2018–2019 burden variables. The findings were consistent with the main analysis: in HICs, diabetes DALY rate remained a significant independent variable associated with RI in all models (MVI model: β = 0.64, 95% CI 0.25–1.03, *P* = .002; SVI model: β = 0.63, 95% CI 0.26–0.99, *P* = .002; Blindness model: β = 0.62, 95% CI 0.22–1.02, *P* = .003; adjusted R^2^ = 0.28–0.29). In LMICs, sensitivity models again yielded no significant associations (all *P* >.2; adjusted R^2^ near zero). These robustness checks support that our main findings are not an artifact of overlapping years of burden and publication measurement (Tables S3 and S4, Supplementary file 2).

####  Visualization of Research Alignment

**Figure 2:**
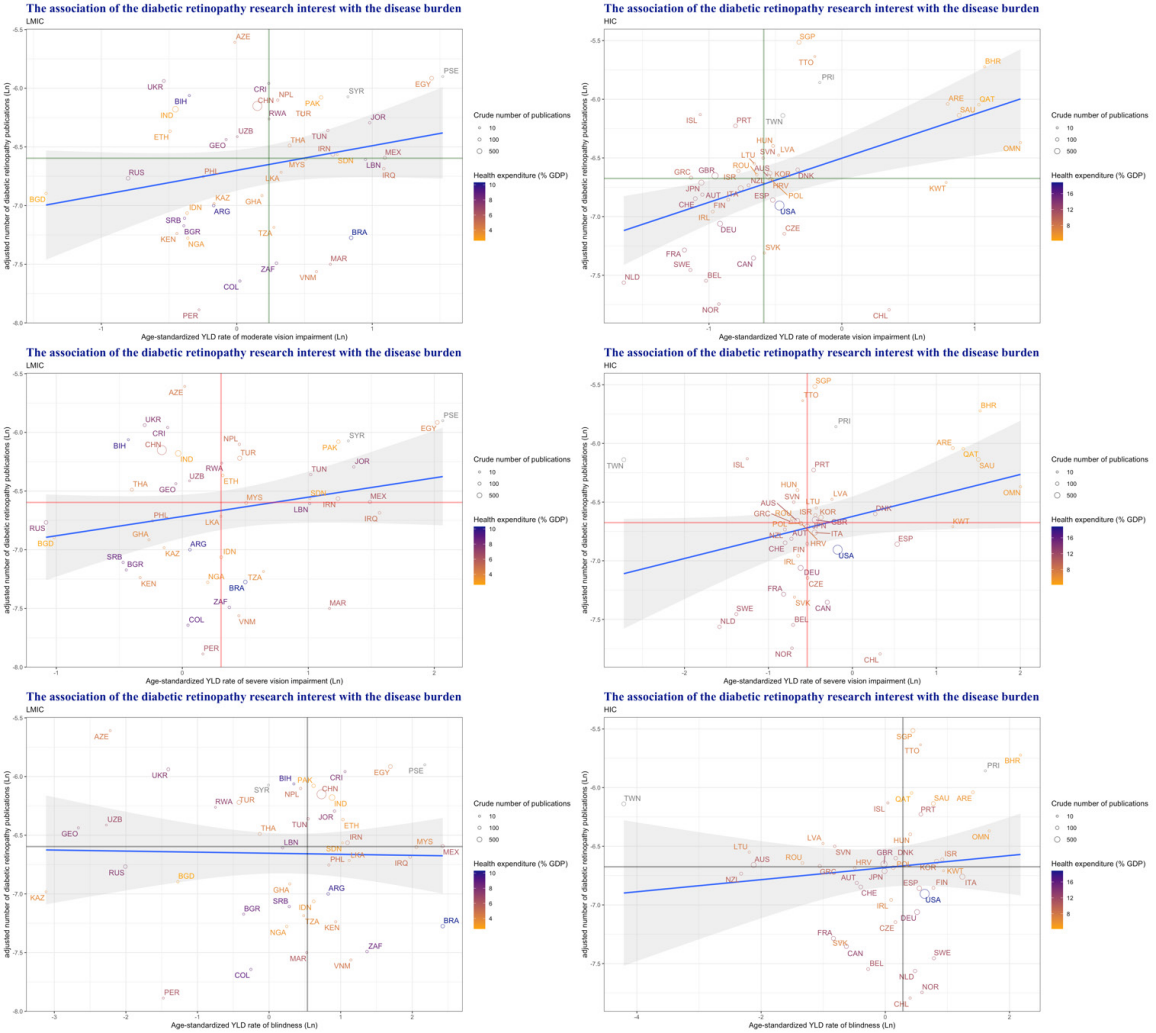
Quadrant Charts Showing the Association of the Diabetic Retinopathy Disease Burden (Age-Standardized YLD Rate in Each Country) in 2018-2019 With its Corresponding RI in 2018-2022; LMICs on the Left and HICs on the Right; Top to Bottom: Moderate Vision Impairment, Severe Vision Impairment, and Blindness, Respectively. Abbreviations: YLD, years lived with disability; GDP, gross domestic product.* The perpendicular lines to the x and y axes represent the median value.

 The relationship between disease burden and RI is visualized in the quadrant plots presented in Figure 2. For HICs, these plots show a discernible, albeit moderate to low, positive trend, with many countries clustering in the “Low burden, Low interest” and “High burden, High interest” quadrants. For LMICs, the plots show a wide and pattern-less scattering of countries across all four quadrants, visually confirming the lack of correlation between research focus and disease burden.

 The heatmaps in Figure S4 further illustrate this misalignment at the country level. Many LMICs, including Iraq, Tanzania, Morocco, and Viet Nam are consistently categorized as “High burden, Low interest” (red), whereas HICs show more varied and often better-aligned profiles (first classification framework based on the concordance of relative DR burden with relative publication output in the field of medicine).

**Figure 3:**
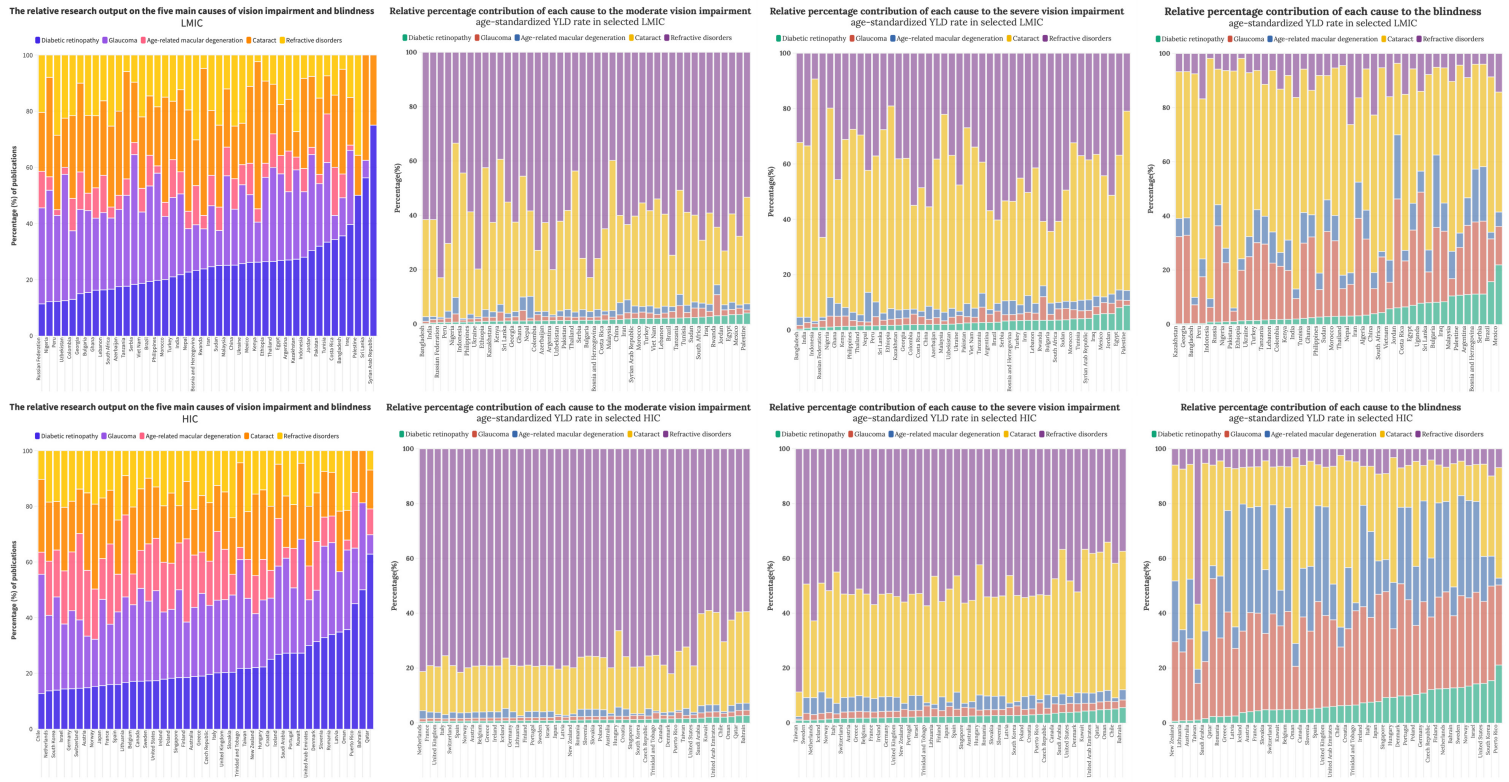
Comparative Alignment of Diabetic Retinopathy Research Output and Disease Burden Relative to Other Major Ocular Diseases Across Country Income Groups (2018–2022). Each row corresponds to a country income group: LMICs on top, and HICs on the bottom. Within each row, the first panel presents the proportional number of publications across five leading ocular diseases (under-corrected refractive error, cataract, age-related macular degeneration, glaucoma, and DR) each represented by a distinct color to differentiate research domains. The second to fourth panels depict the proportional national burden of these same diseases, measured as age-standardized YLD for moderate vision impairment, severe vision impairment, and blindness, respectively. Countries are ordered ascendingly by their proportional publication output on DR within each income group. Abbreviations: HIC, high-income country; LMIC, low- and middle-income country; YLD, years lived with disability; DR, diabetic retinopathy.

 Finally, Figure 3 contextualizes DR research within the broader field of ophthalmology by comparing its publication and burden share against four other leading causes of vision loss. This relative analysis reveals national research priorities within the specialty, highlighting instances where a country’s focus on DR may be high relative to other eye diseases, even if its overall RI appears low when compared to the entire medical field (second classification framework based on the concordance of relative DR burden with relative publication output in ophthalmology field). To conclude, the specific categorization for each country under both frameworks is detailed in Table S5 (Supplementary file 2).

## Discussion

 This study aimed to explore how the national burden of DR, socioeconomic factors, and the RI through DR intersect in LMICs and HICs. Our analysis demonstrated a clear divergence between income groups: while HICs showed a low to moderate bivariate association between DR burden and RI, this was better explained by the overall diabetes burden in adjusted models. Conversely, research patterns in LMICs showed no correlation with DR or diabetes burden in any model. The persistent lack of association in LMICs, even after controlling for these same factors suggests a probable disconnect between local disease burden and national DR research activity. This finding aligns with previous studies investigating the relationship between disease burden and research output,^18,29^ indicating that global research on specific diseases has focused mostly on HIC needs (more prevalent diseases in HICs)^44^ or has been predominantly a derivative of the global market for treatment or large pharmaceutical incentives.^18,44^ This observed disconnect in LMICs may be influenced by systemic factors including indexing biases in international databases, the dominance of HIC-led funding agendas, and limited domestic research infrastructure.

 This gap, although highly speculative, may be driven by a chronic shortage of domestic funding and political prioritization. Many LMICs have historically neglected to allocate significant resources to R&D, viewing it as a long-term expense with delayed rewards.^45^ For instance, despite a pledge made in 2007, no African Union member has reached the goal of investing at least 1% of its GDP in R&D.^46^ This financial and political gap forces local universities and researchers to rely almost entirely on external funding from HICs, which often bring with them priorities that may not align with the health challenges faced locally.

 The nearly identical mean RI values observed between LMICs and HICs may suggest that both groups of countries may be following similar global research patterns rather than reflecting LMIC-specific under-prioritization. This pattern implies that research on DR could be driven by international research agendas, funding flows, and infrastructure availability, rather than by country-specific disease burden.

 Regarding socioeconomic factors, as expected, HICs with higher health expenditures per capita and, in parallel, those allocating a greater percentage of their GDP to health systems, those with greater attention to R&D, and those with higher HDIs generated a greater crude number of DR publications. However, the higher these values are among HICs, the lower the RI is for DR research. One possible explanation of this counterintuitive finding could be that HICs are increasingly investing in emerging research areas, which might partially account for the lower relative focus on DR. Although RI partially aligns with disease burden in HICs, this finding likely does not reflect mismatched priorities or resource allocation within health systems. Rather, it implies a strategic expansion of their research portfolios, a consequence of technological expertise, matured knowledge of disease, and robust market forces that attract research attention to new, profitable frontiers. Nevertheless, this negative correlation might also be partly explained by a denominator effect: wealthier HICs typically produce a much larger total volume of medical and health research, so DR publications may constitute a smaller proportion of their overall scientific output even when the absolute number of DR studies is higher. Such patterns were absent among LMICs.

 We also conducted an additional analysis to elucidate the potential factors influencing the interaction between disease burden and research efforts in both HICs and LMICs, both generally and on a country-specific basis. Our focus was to demonstrate the alignment between overall research trends and the significant drivers of eye-related disease burden within each nation (Figure 3). This approach aims to provide policy-makers with more precise insights from the patterns depicted in quadrant plots (Figure 2). The findings from these analyses are summarized in Table S4, wherein each country is categorized with two descriptors indicating the degree of alignment between disease burden and research efforts under two distinct frameworks of whole medicine (Figure 2) and ophthalmology research (Figure 3). While countries with matching category colors are self-explanatory (25 out of 44 countries, 57% in both HIC and LMIC categories), further explanation is needed for the remainder with 12 different scenarios. For example, Sri Lanka exhibits a state of “Low interest, High burden” relative to the broader medical and life sciences domain among LMICs but presents an inverse scenario in the realm of eye research. This discrepancy likely stems from a disproportionate lack of research focused on eye diseases compared to other health-related areas, despite a relatively high level of interest in DR among individuals with eye disorders. Another example is Singapore, which has a high burden of DR both intrinsically and among eye disorders. Apparently, despite allocating enough percentage of their total research capacity to address their domestic needs for DR research, this topic is of relatively low interest, taking into account only eye diseases, which could be attributed to disproportionately high research output related to other ocular disorders. The other scenarios could be described as such.

 To the best of our knowledge, this is the first bibliometric study in the literature focusing on the differences between LMICs and HICs in DR research, as well as on the disease burden. Regarding the national-level burden of disease, the time interval sufficient to observe the true effects of the hypothetical change in research policies has not been well known until recently.^18^ Previous studies have used a variety of “lag times,” suggesting and ranging from an almost immediate paradigm shift for conference papers,^47^ six months for accelerated peer-review process in the COVID-19 era,^29^ a single year for measuring the association between the burden of disease and research output,^48^ a two-year interval,^18^ and a 5-year cumulative research output including and following the year of which the disease burden data were collected.^44^ Therefore, we decided to use a five-year interval, using the average disease burden data from the first two years. This approach may be most convenient for reducing the possible randomness of using single-year disease burden data, allowing for relatively sufficient time for the research patterns to respond to the burden, possibly in the short term for narrative and systematic reviews and in the long term for original articles (animal studies, randomized controlled trials, etc). Furthermore, we utilized a second classification framework (Figure 3) comparing DR research against other major ocular diseases to ensure that the ‘denominator effect’ of a country’s total medical output did not obscure high-priority research within the ophthalmology specialty.

###  Limitations and Implications for Future Work

 This exploratory study has a diverse set of limitations that will benefit from further research. First, DALY, and YLD as a component of it, are only one of the multiple aspects of estimating disease burden, as estimates based on other assumptions or considering the concept of health needs or well-being might result in different inferences.^49,50^

 Second, we used a retrospective perspective, focusing mainly on the burden of disease (DALY and YLD rates). A more delicate approach would also consider the DR risk factors in a prospective manner. This would be more beneficial for future policy-making. Moreover, our use of DR YLD rates primarily captures the burden of symptomatic vision impairment and blindness. This may not fully reflect research efforts directed toward early-stage screening, prevention, or asymptomatic pathophysiology.

 Third, our bibliometric data are based on a single database (Scopus) and are still more suitable than other databases, such as Web of Science, which has been shown to underrepresent LMICs research.^51^ Moreover, despite our attempts to adapt a validation process for the search strategy, a lack of manual abstract/full text review of the retrieved records leaves a margin for residual false negatives/positives. In addition, the RI, an index defined for the purposes of this study, introduced here takes into account the ratio of the number of peer-reviewed DR publications in the field of medical/life sciences to the whole output of that field in 2018-2022. This approach resulted in a 12% loss in all the retrieved records (exclusively subject areas other than medical/life sciences) and added to the uncertainty of results considering different possible adjustment methods, some of which were tested by the authors and yielded comparable results at the end (data not available). Moreover, we did not differentiate between original research articles and reviews (or among subtypes of research articles), which may introduce bias in accurately reflecting each country’s true research effort and interest in the DR field.

 The national burdens of disease data are only for two consecutive years, with values too close that do not provide sufficient change to interpret a causal effect of health burden on research output. Moreover, as mentioned before, no predetermined time period has been defined to anticipate a change in research output after changing the research policies.

 The simple classification of countries into four possible relative burden/RI categories and the use of two different classification systems, one based on the DR RIs of countries and the other based on the relative RIs from the perspective of ophthalmology research, are helpful only for identifying general patterns. In addition, there is no clearly defined cutoff value to show poor, acceptable, or good concordance of the axes in a four-quadrant graph^52^; the cutoff varies on the basis of the nature of the study and are often arbitrarily chosen. Therefore, the median values were utilized as the best options. Moreover, we did not include the estimates of how far the country values were located from the median of each axis in our final labeling, which can add additional uncertainty to the conclusions. Thus, these general patterns should not be interpreted in a deterministic manner.^44^ Future studies could build on this work by applying bootstrapping or uncertainty-based approaches to test the robustness of country positions and to develop standardized frameworks for similar bibliometric-burden analyses.

 As multiple correlations were tested, the possibility of type I error should be considered. Although formal correction for multiple testing was not applied, in line with common practice in exploratory bibliometric studies, *P* values near the conventional threshold (0.03–0.05) should be interpreted with caution. The analyses are intended to identify general patterns rather than provide definitive inferential conclusions.

 While our results are compatible with anecdotal support from researchers across the literature, we recognize the constraints we face in drawing causal inferences from these data, particularly due to the ecological nature of this study. With limited understanding of the contextual influences shaping our findings and given the considerable differences observed between countries, we invite feedback and open dialog on our findings from the global health research community, particularly from those whose voices are less represented in this field.

## Conclusions

 Our analysis indicates a potential mismatch in the alignment of research efforts with the national DR burden of disease, particularly in LMICs. To address global health disparities in DR research, we recommend implementing strategies to promote coordinated international research efforts supported by HICs and to support local research initiatives in underprivileged areas where health knowledge is most crucial.

## Disclosure of artificial intelligence (AI) use

 Not applicable.

## Ethical issues

 This study was reviewed and approved by Research Ethics Committees of School of Medicine - Tehran University of Medical Sciences with the approval number: IR.TUMS.MEDICINE.REC.1401.674, dated 2023-01-16.

## Conflicts of interest

 Authors declare that they have no conflicts of interest.

## Availability of data and materials

 The burden of disease data used for analysis in this study is available from https://vizhub.healthdata.org/gbd-results/ portal. The other datasets used and/or analyzed during the current study are available from the corresponding author(s) on reasonable request.

## Supplementary files


Supplementary file 1. Fnal Search Strategy.

Supplementary file 2 contains and Tables S1-S5.

Supplementary file 3 contains Figures S1-S4.
