# Supplementary material for: Alignment of Research Efforts With the Diabetic Retinopathy Burden of Disease and Socioeconomic Factors: An Analytical Bibliometric Study
Source: Int J Health Policy Manag. 2026 Apr 11;15:9345. doi: 10.34172/ijhpm.9345 (PMC13338732; doi:10.34172/ijhpm.9345)
Supplement: Supplementary file 1 — Fnal Search Strategy. [file ijhpm-15-9345-s001.pdf]

Article title: Alignment of Research Efforts With the Diabetic Retinopathy Burden of Disease and Socioeconomic Factors: An Analytical Bibliometric Study

Journal name: International Journal of Health Policy and Management (IJHPM)

Authors' information: Farbod Semnani[1,2], Seyed Sahab Aarabi[1,2], Kiana Hassanpour[3], Payam Kabiri[4], Mojtaba Sedaghat[5]*, Amirhossein Takian[6,7,8]*

[1]National Center for Health Insurance Research, Tehran, Iran.

[2]School of Medicine, Tehran University of Medical Sciences (TUMS), Tehran, Iran.

[3]Ophthalmic Research Center, Research Institute for Ophthalmology and Vision Science, Shahid Beheshti University of Medical Sciences, Tehran, Iran.

[4]Department of Biostatistics and Epidemiology, School of Public Health, Tehran University of Medical Sciences (TUMS), Tehran, Iran.

[5]Department of Community Medicine, Faculty of Medicine, Tehran University of Medical Sciences, Tehran, Iran.

[6]Department of Global Health and Public Policy, School of Public Health, Tehran University of Medical Sciences (TUMS), Tehran, Iran.

[7]Department of Health Management, Policy and Economics, School of Public Health, Tehran University of Medical Sciences (TUMS), Tehran, Iran.

[8]Health Equity Research Centre (HERC), Tehran University of Medical Sciences (TUMS), Tehran, Iran.

***Correspondence to:** Mojtaba Sedaghat; Email: sedaghat.dr@gmail.com & Amirhossein Takian; Email: takiana@gmail.com

Citation: Semnani F, Aarabi SS, Hassanpour K, Kabiri P, Sedaghat M, Takian A. Alignment of research efforts with the diabetic retinopathy burden of disease and socioeconomic factors: an analytical bibliometric study. Int J Health Policy Manag. 2026;15:9345. doi:10.34172/ijhpm.9345

Supplementary file 1. Fnal Search Strategy

1. Diabetic Retinopathy (DR):

TITLE-ABS ((diabet* W/5 retinopath*) OR (diabet* W/5 "macular edema") OR (diabet* W/5 maculopath*)) AND (PUBYEAR > 2017 AND PUBYEAR < 2023) AND NOT TITLE ("without diabetic retinopath*" OR "with no diabetic retinopath*" OR "without proliferative diabetic retinopath*" OR "without non-proliferative diabetic retinopath*" OR "without nonproliferative diabetic retinopath*" OR "without diabetic maculopath*" OR "with no diabetic maculopath*" OR "without macular edema" OR "with no macular edema" OR "without diabetes" AND NOT ("with diabetic retinopath*" OR "with diabetic maculopath*" OR "with proliferative diabetic retinopath" OR "with non-proliferative diabetic retinopath" OR "with nonproliferative diabetic retinopath" OR "with and without diabetic retinopath*" OR "with and without diabetic maculopath*" OR "with and without macular edema" OR "with versus without diabetic retinopath*" OR "with versus without diabetic maculopath*" OR "with versus without diabetic macular edema" OR "with and without proliferative diabetic retinopath" OR "with and without non-proliferative diabetic retinopath" OR "with and without nonproliferative diabetic retinopath" OR "with versus without proliferative diabetic retinopath" OR "with versus without non-proliferative diabetic retinopath" OR "with versus without nonproliferative diabetic retinopath")) AND NOT (TITLE (("age-related macular degeneration" OR "age related macular degeneration" OR amd OR "age related maculopath*" OR "age-related maculopath*" OR "choroidal neovascularization" OR "choroidal neovascular membrane*" OR "geographic atrophy*") OR (cataract* OR "capsule W/5 opac*" OR "lens W/5 opac*" OR pseudoaphak*) OR (glaucoma OR "open-angle glaucoma" OR "primary open-angle glaucoma" OR oag OR poag OR "normal tension glaucoma" OR ntg OR "angle closure glaucoma" OR "pseudoexfoliative glaucoma" OR "secondary glaucoma" OR "pigmentary glaucoma" OR "juvenile glaucoma" OR "infantile glaucoma" OR "aphakic glaucoma" OR "phacogenic glaucoma" OR "ciliary block glaucoma" OR "neovascular glaucoma" OR "drug-induced glaucoma") OR (aniseikon* OR anisometrop* OR astigmat* OR "Corneal Wavefront Aberration" OR hyperopi* OR myopi* OR presbyop* OR emmetrop* OR ("refractive W/5 disorder*") OR ("refractive W/5 error*") OR ametrop*) OR ("cardiovascular disease*" OR "kidney disease*" OR "leg ulcer*" OR amputation OR stroke OR nephropath* OR neuropath* OR "heart attack" OR stroke OR cardio* OR renal OR nephro* OR "heart disease*" OR macrovascular OR "myocard* infarct*" OR "foot ulcer*" OR "renal disease" OR "coronary artery disease" OR angiopath*) OR (covid-19 OR "COVID 19") OR ("adie’s pupil" OR strabismus OR albinism OR alzheimer OR dementia OR amblyopia OR anisocoria OR aphakia OR "arcus senilis" OR astigmatism OR keratitis OR "bell’s palsy" OR "black eye" OR blepharitis OR "tear duct" OR "branch retinal vein occlusion" OR cancer OR "carotid artery disease" OR cellulitis OR "central retinal vein occlusion" OR crvo OR "central serous chorioretinopath*" OR stye OR chalazia OR "choroidal neovascular membrane*" OR coloboma OR "corneal abrasion" OR "contact lens" OR "corneal dystroph*" OR "cytomegalovirus retinitis" OR "corneal laceration" OR "dry eye" OR "detached retina" OR drusen OR ectropion OR endophthalmitis OR entropion OR "eye allerg*" OR "epiretinal membrane" OR lymphoma OR fuchs OR "giant cell arthritis" OR conjunctivitis OR hemangioma OR shingles OR hyphema OR "hypertensive retinopath*" OR heterochromia OR histoplasmosis OR "intracranial hypertension" OR "Irvine Gass" OR uveit* OR "juvenile macular dystroph*" OR keratoconus OR "lattice degeneration" OR marfan OR migraine OR milia OR "Myasthenia Gravis" OR "Microvascular Cranial Nerve Palsy" OR nevus OR nystagmus OR "Neuropathic Corneal Pain"

OR melanoma OR "Orbital Fracture" OR rosacea OR "Pigment Dispersion Syndrome" OR "Sickle cell" OR pterygium OR pinguecula OR "Posterior Vitreous Detachment" OR ptosis OR "Pseudoexfoliation Syndrome" OR "Retinal Artery Occlusion" OR "Retinal Vein Occlusion" OR "Retinitis Pigmentosa" OR "Retinoblastoma" OR "Retinopathy of Prematurity" OR "River Blindness" OR "Sjogren’s Syndrome" OR scleritis OR "Subconjunctival Hemorrhage" OR "Thyroid Eye Disease" OR "thalassem*" OR trachoma OR toxoplasmosis OR "Usher Syndrome" OR "Vitreomacular Traction" OR "Vitamin A Deficiency" OR xanthelasma)) AND NOT TITLE ((diabet* W/5 retinopath*) OR (diabet* W/5 "macular edema") OR (diabet* W/5 maculopath*)))

2. ***Cataract:***

TITLE-ABS (cataract* OR "capsule W/5 opac*" OR "lens W/5 opac*" OR pseudoaphak*) AND (PUBYEAR > 2017 AND PUBYEAR < 2023) AND NOT (TITLE (("age-related macular degeneration" OR "age related macular degeneration" OR amd OR "age related maculopath*" OR "age-related maculopath*" OR "choroidal neovascularization" OR "choroidal neovascular membrane*" OR "geographic atrophy*") OR ((diabet* W/5 retinopath*) OR (diabet* W/5 "macular edema") OR (diabet* W/5 maculopath*)) OR (glaucoma OR "open-angle glaucoma" OR "primary open-angle glaucoma" OR oag OR poag OR "normal tension glaucoma" OR ntg OR "angle closure glaucoma" OR "pseudoexfoliative glaucoma" OR "secondary glaucoma" OR "pigmentary glaucoma" OR "juvenile glaucoma" OR "infantile glaucoma" OR "aphakic glaucoma" OR "phacogenic glaucoma" OR "ciliary block glaucoma" OR "neovascular glaucoma" OR "drug-induced glaucoma") OR (aniseikon* OR anisometrop* OR astigmat* OR "Corneal Wavefront Aberration" OR hyperopi* OR myopi* OR presbyop* OR emmetrop* OR ("refractive W/5 disorder*") OR ("refractive W/5 error*") OR ametrop*) OR (covid-19 OR "COVID 19") OR ("adie's pupil" OR strabismus OR albinism OR alzheimer OR dementia OR amblyopia OR anisocoria OR aphakia OR "arcus senilis" OR astigmatism OR keratitis OR "bell's palsy" OR "black eye" OR blepharitis OR "tear duct" OR "branch retinal vein occlusion" OR cancer OR "carotid artery disease" OR cellulitis OR "central retinal vein occlusion" OR crvo OR "central serous chorioretinopath*" OR stye OR chalazia OR "choroidal neovascular membrane*" OR coloboma OR "corneal abrasion" OR "contact lens" OR "corneal dystroph*" OR "cytomegalovirus retinitis" OR "corneal laceration" OR "dry eye" OR "detached retina" OR drusen OR ectropion OR endophthalmitis OR entropion OR "eye allerg*" OR "epiretinal membrane" OR lymphoma OR "foreign body" OR fuchs OR "giant cell arthritis" OR conjunctivitis OR hemangioma OR shingles OR hyphema OR "hypertensive retinopath*" OR heterochromia OR histoplasmosis OR "intracranial hypertension" OR "Irvine Gass" OR uveit* OR "juvenile macular dystroph*" OR keratoconus OR "lattice degeneration" OR "macular edema" OR marfan OR migraine OR milia OR "Myasthenia Gravis" OR "Microvascular Cranial Nerve Palsy" OR nevus OR nystagmus OR "Neuropathic Corneal Pain" OR melanoma OR "Orbital Fracture" OR rosacea OR "Pigment Dispersion Syndrome" OR "Sickle cell" OR pterygium OR pinguecula OR "Posterior Vitreous Detachment" OR ptosis OR "Pseudoexfoliation Syndrome" OR "Retinal Artery Occlusion" OR "Retinal Vein Occlusion" OR "Retinitis Pigmentosa" OR "Retinoblastoma" OR "Retinopathy of Prematurity" OR "River Blindness" OR "Sjogren's Syndrome" OR scleritis OR "Subconjunctival Hemorrhage" OR "Thyroid Eye Disease" OR "thalassem*" OR trachoma OR toxoplasmosis OR "Usher Syndrome" OR "Vitreomacular Traction" OR "Vitamin A Deficiency" OR xanthelasma)) AND NOT TITLE (cataract* OR "capsule W/5 opac*" OR "lens W/5 opac*" OR pseudoaphak*))

3. Age-related Macular Degeneration(AMD):

TITLE-ABS ("age-related macular degeneration" OR "age related macular degeneration" OR "age related maculopath*" OR "age-related maculopath*" OR "choroidal neovascularization" OR "choroidal neovascular membrane*" OR "geographic atrophy*") AND (PUBYEAR > 2017 AND PUBYEAR < 2023) AND NOT (TITLE ((glaucoma OR "open-angle glaucoma" OR "primary open-angle glaucoma" OR oag OR poag OR "normal tension glaucoma" OR ntg OR "angle closure glaucoma" OR "pseudoexfoliative glaucoma" OR "secondary glaucoma" OR "pigmentary glaucoma" OR "juvenile glaucoma" OR "infantile glaucoma" OR "aphakic glaucoma" OR "phacogenic glaucoma" OR "ciliary block glaucoma" OR "neovascular glaucoma" OR "drug-induced glaucoma") OR ((diabet* W/5 retinopath*) OR (diabet* W/5 "macular edema") OR (diabet* W/5 maculopath*)) OR (cataract* OR "capsule W/5 opac*" OR "lens W/5 opac*" OR pseudoaphak*) OR (aniseikon* OR anisometrop* OR astigmat* OR "Corneal Wavefront Aberration" OR hyperopi* OR myopi* OR presbyop* OR emmetrop* OR ("refractive W/5 disorder*") OR ("refractive W/5 error*") OR ametrop*) OR (covid-19 OR "COVID 19") OR ("adie's pupil" OR strabismus OR albinism OR alzheimer OR dementia OR amblyopia OR anisocoria OR aphakia OR "arcus senilis" OR astigmatism OR keratitis OR "bell's palsy" OR "black eye" OR blepharitis OR "tear duct" OR "branch retinal vein occlusion" OR cancer OR "carotid artery disease" OR cellulitis OR "central retinal vein occlusion" OR crvo OR "central serous chorioretinopath*" OR stye OR chalazia OR coloboma OR "corneal abrasion" OR "contact lens" OR "corneal dystroph*" OR "cytomegalovirus retinitis" OR "corneal laceration" OR "dry eye" OR "detached retina" OR drusen OR ectropion OR endophthalmitis OR entropion OR "eye allerg*" OR "epiretinal membrane" OR lymphoma OR "foreign body" OR fuchs OR "giant cell arthritis" OR conjunctivitis OR hemangioma OR shingles OR hyphema OR "hypertensive retinopath*" OR heterochromia OR histoplasmosis OR "intracranial hypertension" OR "Irvine Gass" OR uveit* OR "juvenile macular dystroph*" OR keratoconus OR "lattice degeneration" OR "macular edema" OR marfan OR migraine OR milia OR "Myasthenia Gravis" OR "Microvascular Cranial Nerve Palsy" OR nevus OR nystagmus OR "Neuropathic Corneal Pain" OR melanoma OR "Orbital Fracture" OR rosacea OR "Pigment Dispersion Syndrome" OR "Sickle cell" OR pterygium OR pinguecula OR "Posterior Vitreous Detachment" OR ptosis OR "Pseudoexfoliation Syndrome" OR "Retinal Artery Occlusion" OR "Retinal Vein Occlusion" OR "Retinitis Pigmentosa" OR "Retinoblastoma" OR "Retinopathy of Prematurity" OR "River Blindness" OR "Sjogren's Syndrome" OR scleritis OR "Subconjunctival Hemorrhage" OR "Thyroid Eye Disease" OR "thalassem*" OR trachoma OR toxoplasmosis OR "Usher Syndrome" OR "Vitreomacular Traction" OR "Vitamin A Deficiency" OR xanthelasma)) AND NOT TITLE ("age-related macular degeneration" OR "age related macular degeneration" OR "age related maculopath*" OR "age-related maculopath*" OR "choroidal neovascularization" OR "choroidal neovascular membrane*" OR "geographic atrophy*"))

4. Refractive disorders:

TITLE-ABS (aniseikon* OR anisometrop* OR astigmat* OR "Corneal Wavefront Aberration" OR hyperopi* OR myopi* OR presbyop* OR emmetrop* OR ("refractive W/5 disorder*") OR ("refractive W/5 error*") OR ametrop*) AND (PUBYEAR > 2017 AND PUBYEAR < 2023) AND NOT (TITLE ((glaucoma OR "open-angle glaucoma" OR "primary open-angle glaucoma" OR oag OR poag OR "normal tension glaucoma" OR ntg OR "angle closure glaucoma" OR "pseudoexfoliative glaucoma" OR "secondary glaucoma" OR "pigmentary glaucoma" OR "juvenile glaucoma" OR "infantile glaucoma" OR "aphakic glaucoma" OR "phacogenic glaucoma" OR "ciliary block glaucoma" OR "neovascular glaucoma" OR "drug-induced glaucoma") OR ((diabet* W/5 retinopath*) OR (diabet* W/5 "macular edema") OR (diabet* W/5 maculopath*)) OR (cataract* OR "capsule W/5 opac*" OR "lens W/5 opac*" OR pseudoaphak*) OR ("age-related macular degeneration" OR "age related macular degeneration" OR "age related maculopath*" OR "age-related maculopath*" OR "choroidal neovascularization" OR "choroidal neovascular membrane*" OR "geographic atrophy*") OR (covid-19 OR "COVID 19") OR ("adie's pupil" OR strabismus OR albinism OR alzheimer OR dementia OR amblyopia OR anisocoria OR aphakia OR "arcus senilis" OR keratitis OR "bell's palsy" OR "black eye" OR blepharitis OR "tear duct" OR "branch retinal vein occlusion" OR cancer OR "carotid artery disease" OR cellulitis OR "central retinal vein occlusion" OR crvo OR "central serous chorioretinopath*" OR stye OR chalazia OR coloboma OR "corneal abrasion" OR "contact lens" OR "corneal dystroph*" OR "cytomegalovirus retinitis" OR "corneal laceration" OR "dry eye" OR "detached retina" OR "retinal detachment" OR drusen OR ectropion OR endophthalmitis OR entropion OR "eye allerg*" OR "epiretinal membrane" OR lymphoma OR "foreign body" OR fuchs OR "giant cell arthritis" OR conjunctivitis OR hemangioma OR shingles OR hyphema OR "hypertensive retinopath*" OR heterochromia OR histoplasmosis OR "intracranial hypertension" OR "Irvine Gass" OR uveit* OR "juvenile macular dystroph*" OR keratoconus OR "lattice degeneration" OR "macular edema" OR marfan OR migraine OR milia OR "Myasthenia Gravis" OR "Microvascular Cranial Nerve Palsy" OR nevus OR nystagmus OR "Neuropathic Corneal Pain" OR melanoma OR "Orbital Fracture" OR rosacea OR "Pigment Dispersion Syndrome" OR "Sickle cell" OR pterygium OR pinguecula OR "Posterior Vitreous Detachment" OR ptosis OR "Pseudoexfoliation Syndrome" OR "Retinal Artery Occlusion" OR "Retinal Vein Occlusion" OR "Retinitis Pigmentosa" OR "Retinoblastoma" OR "Retinopathy of Prematurity" OR "River Blindness" OR "Sjogren's Syndrome" OR scleritis OR "Subconjunctival Hemorrhage" OR "Thyroid Eye Disease" OR "thalassem*" OR trachoma OR toxoplasmosis OR "Usher Syndrome" OR "Vitreomacular Traction" OR "Vitamin A Deficiency" OR xanthelasma)) AND NOT TITLE (aniseikon* OR anisometrop* OR astigmat* OR "Corneal Wavefront Aberration" OR hyperopi* OR myopi* OR presbyop* OR emmetrop* OR ("refractive W/5 disorder*") OR ("refractive W/5 error*") OR ametrop*))

5. Glaucoma:

TITLE-ABS (aniseikon* OR anisometrop* OR astigmat* OR "Corneal Wavefront Aberration" OR hyperopi* OR myopi* OR presbyop* OR emmetrop* OR ("refractive W/5 disorder*") OR ("refractive W/5 error*") OR ametrop*) AND (PUBYEAR > 2017 AND PUBYEAR < 2023) AND NOT (TITLE ((glaucoma OR "open-angle glaucoma" OR "primary open-angle glaucoma" OR oag OR poag OR "normal tension glaucoma" OR ntg OR "angle closure glaucoma" OR "pseudoexfoliative glaucoma" OR "secondary glaucoma" OR "pigmentary glaucoma" OR "juvenile glaucoma" OR "infantile glaucoma" OR "aphakic glaucoma" OR "phacogenic glaucoma" OR "ciliary block glaucoma" OR "neovascular glaucoma" OR "drug-induced glaucoma") OR ((diabet* W/5 retinopath*) OR (diabet* W/5 "macular edema") OR (diabet* W/5 maculopath*)) OR (cataract* OR "capsule W/5 opac*" OR "lens W/5 opac*" OR pseudoaphak*) OR ("age-related macular degeneration" OR "age related macular degeneration" OR "age related maculopath*" OR "age-related maculopath*" OR "choroidal neovascularization" OR "choroidal neovascular membrane*" OR "geographic atrophy*") OR (covid-19 OR "COVID 19") OR ("adie's pupil" OR strabismus OR albinism OR alzheimer OR dementia OR amblyopia OR anisocoria OR aphakia OR "arcus senilis" OR keratitis OR "bell's palsy" OR "black eye" OR blepharitis OR "tear duct" OR "branch retinal vein occlusion" OR cancer OR "carotid artery disease" OR cellulitis OR "central retinal vein occlusion" OR crvo OR "central serous chorioretinopath*" OR stye OR chalazia OR coloboma OR "corneal abrasion" OR "contact lens" OR "corneal dystroph*" OR "cytomegalovirus retinitis" OR "corneal laceration" OR "dry eye" OR "detached retina" OR "retinal detachment" OR drusen OR ectropion OR endophthalmitis OR entropion OR "eye allerg*" OR "epiretinal membrane" OR lymphoma OR "foreign body" OR fuchs OR "giant cell arthritis" OR conjunctivitis OR hemangioma OR shingles OR hyphema OR "hypertensive retinopath*" OR heterochromia OR histoplasmosis OR "intracranial hypertension" OR "Irvine Gass" OR uveit* OR "juvenile macular dystroph*" OR keratoconus OR "lattice degeneration" OR "macular edema" OR marfan OR migraine OR milia OR "Myasthenia Gravis" OR "Microvascular Cranial Nerve Palsy" OR nevus OR nystagmus OR "Neuropathic Corneal Pain" OR melanoma OR "Orbital Fracture" OR rosacea OR "Pigment Dispersion Syndrome" OR "Sickle cell" OR pterygium OR pinguecula OR "Posterior Vitreous Detachment" OR ptosis OR "Pseudoexfoliation Syndrome" OR "Retinal Artery Occlusion" OR "Retinal Vein Occlusion" OR "Retinitis Pigmentosa" OR "Retinoblastoma" OR "Retinopathy of Prematurity" OR "River Blindness" OR "Sjogren's Syndrome" OR scleritis OR "Subconjunctival Hemorrhage" OR "Thyroid Eye Disease" OR "thalassem*" OR trachoma OR toxoplasmosis OR "Usher Syndrome" OR "Vitreomacular Traction" OR "Vitamin A Deficiency" OR xanthelasma)) AND NOT TITLE (aniseikon* OR anisometrop* OR astigmat* OR "Corneal Wavefront Aberration" OR hyperopi* OR myopi* OR presbyop* OR emmetrop* OR ("refractive W/5 disorder*") OR ("refractive W/5 error*") OR ametrop*))
