# Supplementary material for: Alignment of Research Efforts With the Diabetic Retinopathy Burden of Disease and Socioeconomic Factors: An Analytical Bibliometric Study
Source: Int J Health Policy Manag. 2026 Apr 11;15:9345. doi: 10.34172/ijhpm.9345 (PMC13338732; doi:10.34172/ijhpm.9345)
Supplement: Supplementary file 2 — contains and Tables S1-S5. [file ijhpm-15-9345-s002.pdf]

Article title: Alignment of Research Efforts With the Diabetic Retinopathy Burden of Disease and Socioeconomic Factors: An Analytical Bibliometric Study

Journal name: International Journal of Health Policy and Management (IJHPM)

Authors' information: Farbod Semnani[1,2], Seyed Sahab Aarabi[1,2], Kiana Hassanpour[3], Payam Kabiri[4], Mojtaba Sedaghat[5]*, Amirhossein Takian[6,7,8]*

[1]National Center for Health Insurance Research, Tehran, Iran.

[2]School of Medicine, Tehran University of Medical Sciences (TUMS), Tehran, Iran.

[3]Ophthalmic Research Center, Research Institute for Ophthalmology and Vision Science, Shahid Beheshti University of Medical Sciences, Tehran, Iran.

[4]Department of Biostatistics and Epidemiology, School of Public Health, Tehran University of Medical Sciences (TUMS), Tehran, Iran.

[5]Department of Community Medicine, Faculty of Medicine, Tehran University of Medical Sciences, Tehran, Iran.

[6]Department of Global Health and Public Policy, School of Public Health, Tehran University of Medical Sciences (TUMS), Tehran, Iran.

[7]Department of Health Management, Policy and Economics, School of Public Health, Tehran University of Medical Sciences (TUMS), Tehran, Iran.

[8]Health Equity Research Centre (HERC), Tehran University of Medical Sciences (TUMS), Tehran, Iran.

***Correspondence to:** Mojtaba Sedaghat; Email: sedaghat.dr@gmail.com & Amirhossein Takian; Email: takiana@gmail.com

Citation: Semnani F, Aarabi SS, Hassanpour K, Kabiri P, Sedaghat M, Takian A. Alignment of research efforts with the diabetic retinopathy burden of disease and socioeconomic factors: an analytical bibliometric study. Int J Health Policy Manag. 2026;15:9345. doi:10.34172/ijhpm.9345

Supplementary file 2

Table S1. Comparing the number of publications found for the 10 most productive authors in our search with the DR-related publication counts present in their Scopus personal profiles during 2018-2022

Author	Scopus profile (N)	Search (N)
Wong, Tienyin	93	90
Sivaprasad, Sobha	86	85
Bandello, Francesco Maria	67	65
Raman, Rajiv P.G.	65	64
Peto, Tunde	62	59
Wykoff, Charles Clifton	59	54
Sun, Jennifer K.	57	53
Grauslund, Jakob	53	45
Sabanayagam, C.	50	45
Li, Xiaorong	49	44

Table S2. Variance Inflation Factors (VIF) for multivariable linear regression models (2018-2022 – main models)

Category	DR Burden Model	Variable	VIF	Category	DR Burden Model	Variable	VIF
HIC	MVI	MVI	2.03	LMIC	MVI	MVI	1.53
		DB	2.19			DB	1.65
		HDI	2.87			HDI	2.81
		**HEpc (PPP)**	2.39			**HEpc (PPP)**	2.92
		**Mean**	2.37			**Mean**	2.23
	SVI	SVI	1.7		SVI	SVI	1.62
		DB	1.98			DB	1.69
		HDI	2.85			HDI	2.77
		**HEpc (PPP)**	2.39			**HEpc (PPP)**	2.93
		**Mean**	2.23			**Mean**	2.25
	Blindness	Blindness	1.69		Blindness	Blindness	1.57
		DB	2.33			DB	1.73

		HDI	2.98			HDI	2.78
		**HEpc (PPP)**	2.41			**HEpc (PPP)**	3.11
		**Mean**	2.35			**Mean**	2.30

MVI, moderate vision impairment; SVI, severe vision impairment; DB, diabetes burden; HDI, human development index; HEpc (PPP), health expenditure per capita, purchasing power parity ($); HIC, high-income countries; LMIC, low- and middle-income countries; DR, diabetic retinopathy.

Table S3. Spearman's rank correlation coefficient (Rho) of the RI in 2020-2022 period toward DR research with age-standardized DR-attributable YLD rates of MVI, SVI, and blindness (for 2018-2019), and with health expenditure per capita purchasing power parity (PPP), current health expenditure (% of GDP), R & D, and HDI (Sensitivity analysis)

	**MVI**		**SVI**		**Blindness**		**health expenditure per capita (PPP)**		**current health expenditure (% of GDP)**		**R & D**		**HDI**	
**Category**	LMIC	HIC	LMIC	HIC	LMIC	HIC	LMIC	HIC	LMIC	HIC	LMIC	HIC	LMIC	HIC
**Rho (ρ)**	0.24	0.47	0.27	0.31	0.05	0.11	- 0.23	-0.48	- 0.24	-0.59	- 0.06	-0.39	-0.14	-0.26
**P-value**	0.12	0.001	0.08	0.04	0.77	0.47	0.15	0.001	0.13	< 0.001	0.73	0.009	0.36	0.09

MVI, moderate vision impairment; SVI, severe vision impairment; PPP, purchasing power parity; GDP, gross domestic product; R&D, research and development index; HDI, human development index; RI, research interest; YLD, years lived with disability; HIC, high-income countries; LMIC, low- and middle-income countries.

Table S4. Multivariable linear regression analysis of the association between diabetic retinopathy burden indicators (2018-2019) and national research interest in diabetic retinopathy during 2020-2022, stratified by country income level. Covariates included the Human Development Index (HDI), health expenditure per capita (PPP, international dollars), and diabetes burden (Age-standardized DALY rate). (Sensitivity analysis)

Category	DR burden Model	Predictor	β Coefficient	95% CI	p-value	Adjusted R^2
**HIC**	MVI	MVI	-0.08	-0.45 to 0.29	0.65	0.29
		DB	0.64	0.25 to 1.03	0.002	
		HDI	0.33	-0.12 to 0.78	0.15	
		**HEpc (PPP)**	-0.33	-0.75 to 0.08	0.11	
	SVI	SVI	-0.07	-0.41 to 0.27	0.68	0.28
		DB	0.63	0.26 to 0.99	0.002	
		HDI	0.34	-0.11 to 0.78	0.14	
		**HEpc (PPP)**	-0.33	-0.74 to 0.08	0.11	
	Blindness	Blindness	-0.06	-0.42 to 0.30	0.75	0.28
		DB	0.62	0.22 to 1.02	0.003	
		HDI	0.36	-0.10 to 0.82	0.12	
		**HEpc (PPP)**	-0.34	-0.75 to 0.07	0.10	
**LMIC**	MVI	MVI	0.22	-0.17 to 0.61	0.27	0.02

	DB	-0.03	-0.43 to 0.38	0.90	
	HDI	0.10	-0.43 to 0.63	0.71	
	**HEpc (PPP)**	-0.32	-0.86 to 0.22	0.24	
SVI	SVI	0.24	-0.17 to 0.64	0.24	0.02
	DB	-0.04	-0.45 to 0.37	0.85	
	HDI	0.12	-0.41 to 0.65	0.65	
	**HEpc (PPP)**	-0.31	-0.85 to 0.24	0.26	
Blindness	Blindness	-0.07	-0.48 to 0.33	0.72	-0.02
	DB	0.15	-0.28 to 0.57	0.48	
	HDI	0.13	-0.41 to 0.67	0.62	
	**HEpc (PPP)**	-0.31	-0.88 to 0.26	0.28	

MVI, moderate vision impairment; SVI, severe vision impairment; DB, diabetes burden; HDI, human development index; HEpc (PPP), health expenditure per capita, purchasing power parity ($); HIC, high-income countries; LMIC, low- and middle-income countries; DR, diabetic retinopathy; CI, confidence interval

Table S5. The association of the disease burden with the RI toward DR, and its congruence with the association of the DR RI with relative burden among the five major causes of vision impairment and blindness in the selected LMICs and HICs

Low-income country	Scatterplot category(relative to whole field of medicine)*	Stacked bar chart category(relative to ophthalmology field only)*	High-income country	Scatterplot category(relative to whole field of medicine)*	Stacked bar chart category(relative to ophthalmology field only)*
LMIC			HIC		
Argentina			Australia		
Azerbaijan			Austria		
Bangladesh			Bahrain		
Bosnia and Herzegovina			Belgium		
Brazil			Canada		
Bulgaria			Chile		
China			Croatia		
Colombia			Czech Republic		
Costa Rica			Denmark		
Egypt			Finland		
Ethiopia			France		
Georgia			Germany		
Ghana			Greece		

**India**			**Hungary**		
**Indonesia**			**Iceland**		
**Iran**			**Ireland**		
**Iraq**			**Israel**		
**Jordan**			**Italy**		
**Kazakhstan**			**Japan**		
**Kenya**			**Kuwait**		
**Lebanon**			**Latvia**		
**Malaysia**			**Lithuania**		
**Mexico**			**Netherlands**		
**Morocco**			**New Zealand**		
**Nepal**			**Norway**		
**Nigeria**			**Oman**		
**Pakistan**			**Poland**		
**Palestine**			**Portugal**		
**Peru**			**Puerto Rico**		
**Philippines**			**Qatar**		
**Russian Federation**			**Romania**		
**Rwanda**			**Saudi Arabia**		
**Serbia**			**Singapore**		

**South Africa**			**Slovakia**		
**Sri Lanka**			**Slovenia**		
**Sudan**			**South Korea**		
**Syrian Arab Republic**			**Spain**		
**Tanzania**			**Sweden**		
**Thailand**			**Switzerland**		
**Tunisia**			**Taiwan**		
**Turkey**			**Trinidad and Tobago**		
**Ukraine**			**United Arab Emirates**		
**Uzbekistan**			**United Kingdom**		
**Viet Nam**			**United States**		

*The most common category among the three burden estimates: Average age-standardized YLD rates of MVI, SVI, and blindness in each country between 2018-2019

*Green: Low relative RI, Low relative disease burden; Red: Low relative RI, High relative disease burden/ Orange: High relative RI, Low relative disease burden/ Blue: High relative RI, High relative disease burden

HIC, high-income countries; LMIC, low- and middle-income countries.
