# Supplementary material for: Alignment of Research Efforts With the Diabetic Retinopathy Burden of Disease and Socioeconomic Factors: An Analytical Bibliometric Study
Source: Int J Health Policy Manag. 2026 Apr 11;15:9345. doi: 10.34172/ijhpm.9345 (PMC13338732; doi:10.34172/ijhpm.9345)

Article title: Alignment of Research Efforts With the Diabetic Retinopathy Burden of Disease and Socioeconomic Factors: An Analytical Bibliometric Study

Journal name: International Journal of Health Policy and Management (IJHPM)

Authors' information: Farbod Semnani[1,2], Seyed Sahab Aarabi[1,2], Kiana Hassanpour[3], Payam Kabiri[4], Mojtaba Sedaghat[5]*, Amirhossein Takian[6,7,8]*

[1]National Center for Health Insurance Research, Tehran, Iran.

[2]School of Medicine, Tehran University of Medical Sciences (TUMS), Tehran, Iran.

[3]Ophthalmic Research Center, Research Institute for Ophthalmology and Vision Science, Shahid Beheshti University of Medical Sciences, Tehran, Iran.

[4]Department of Biostatistics and Epidemiology, School of Public Health, Tehran University of Medical Sciences (TUMS), Tehran, Iran.

[5]Department of Community Medicine, Faculty of Medicine, Tehran University of Medical Sciences, Tehran, Iran.

[6]Department of Global Health and Public Policy, School of Public Health, Tehran University of Medical Sciences (TUMS), Tehran, Iran.

[7]Department of Health Management, Policy and Economics, School of Public Health, Tehran University of Medical Sciences (TUMS), Tehran, Iran.

[8]Health Equity Research Centre (HERC), Tehran University of Medical Sciences (TUMS), Tehran, Iran.

***Correspondence to:** Mojtaba Sedaghat; Email: sedaghat.dr@gmail.com & Amirhossein Takian; Email: takiana@gmail.com

Citation: Semnani F, Aarabi SS, Hassanpour K, Kabiri P, Sedaghat M, Takian A. Alignment of research efforts with the diabetic retinopathy burden of disease and socioeconomic factors: an analytical bibliometric study. Int J Health Policy Manag. 2026;15:9345. doi:10.34172/ijhpm.9345

Supplementary file 3

Figure S1. The trend of diabetic retinopathy research output in Scopus from 2018-2022. DR, diabetic retinopathy.

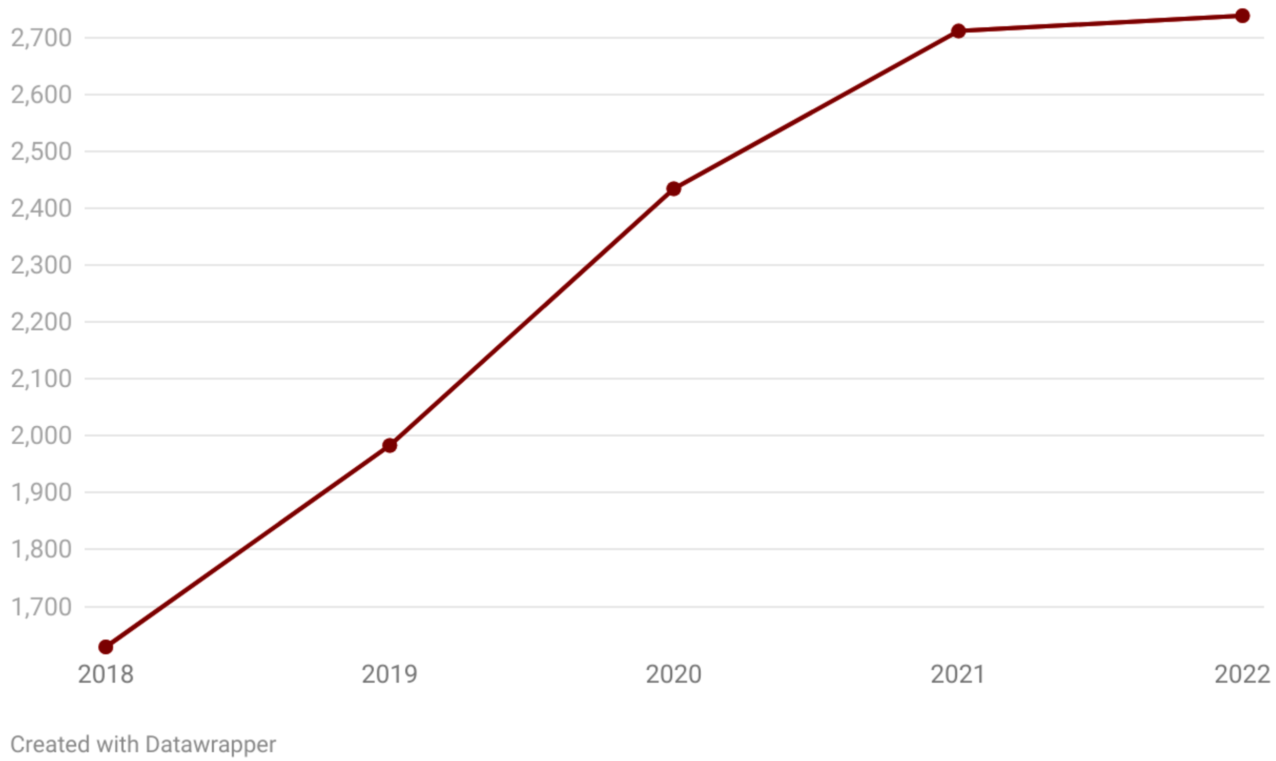

Figure S2. The Global cartogram of the distribution of countries with at least five DR related publications in the medical & life sciences field between 2018-2022.

This figure presents a global cartogram, a map in which the geographic size of countries is distorted to be proportional to their research output in diabetic retinopathy (DR) from 2018-2022. Countries with a higher number of publications appear larger, while those with fewer publications shrink.

The figure confirms that DR research is highly concentrated in a few regions. North America (especially the USA), Western Europe, and East Asia (particularly China) are dramatically enlarged, indicating high publication volumes. On the other hand, entire regions such as Africa and South America are shrunk to the point of being almost invisible on the map, reflecting their markedly lower research productivity in this field.

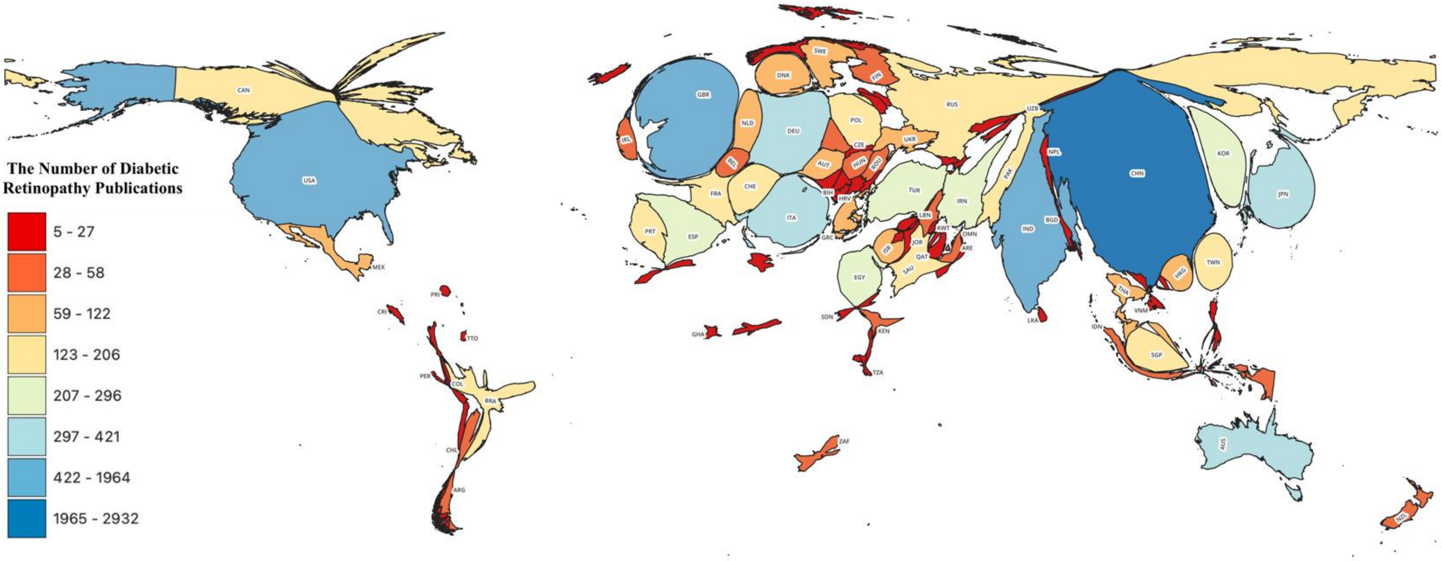

Figure S3. Choropleth map depicting the relative burden of DR according to the countries with at least five DR related publications between 2018-2022.YLD, years lived with disability; MVI, moderate vision impairment; SVI, severe vision impairment.

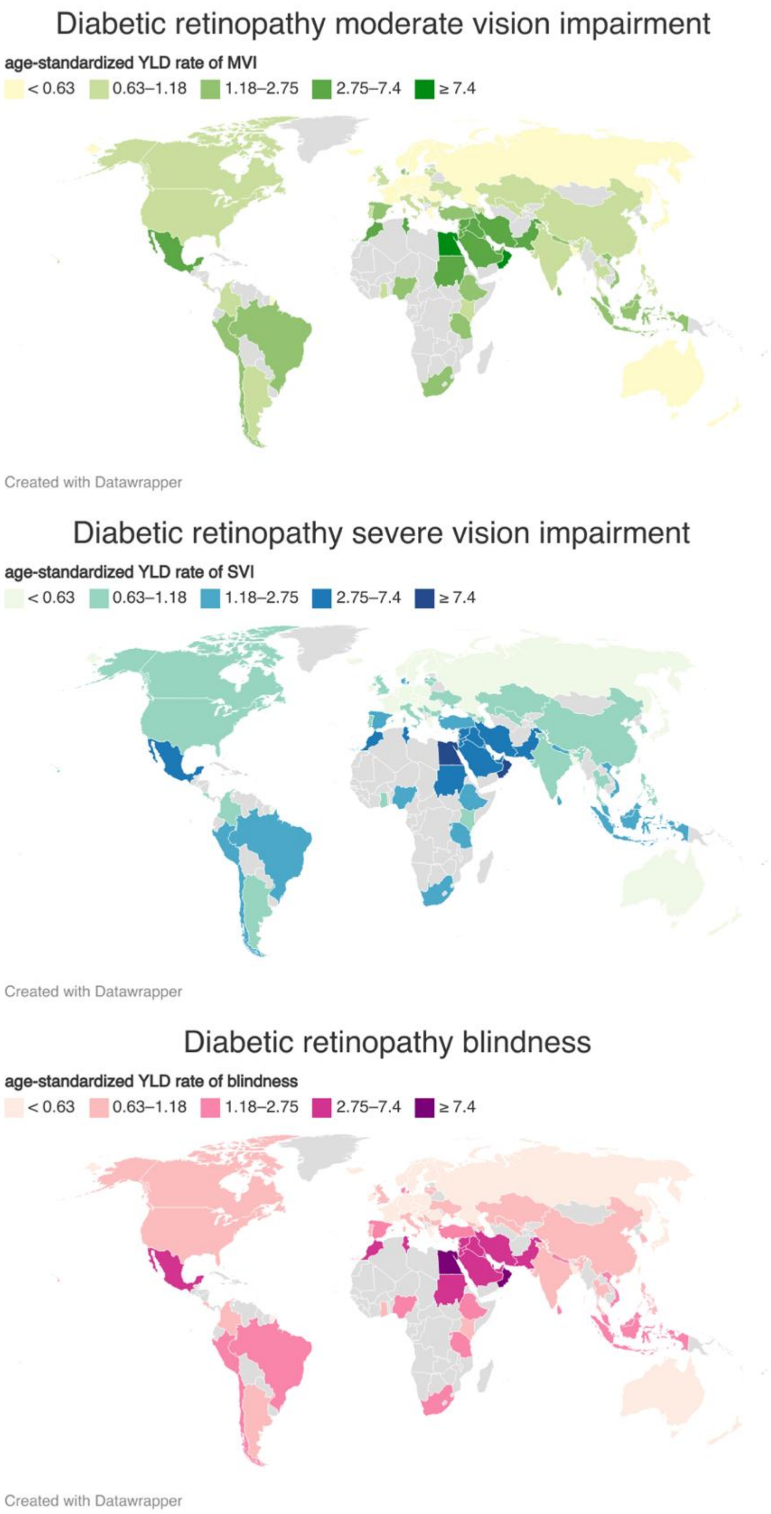

Figure S4. Heatmaps depicting the relative alignment of research interest of different countries with their disease burden. HIC, high-income countries; LMIC low- and middle-income countries; MVI, moderate vision impairment; SVI, severe vision impairment.

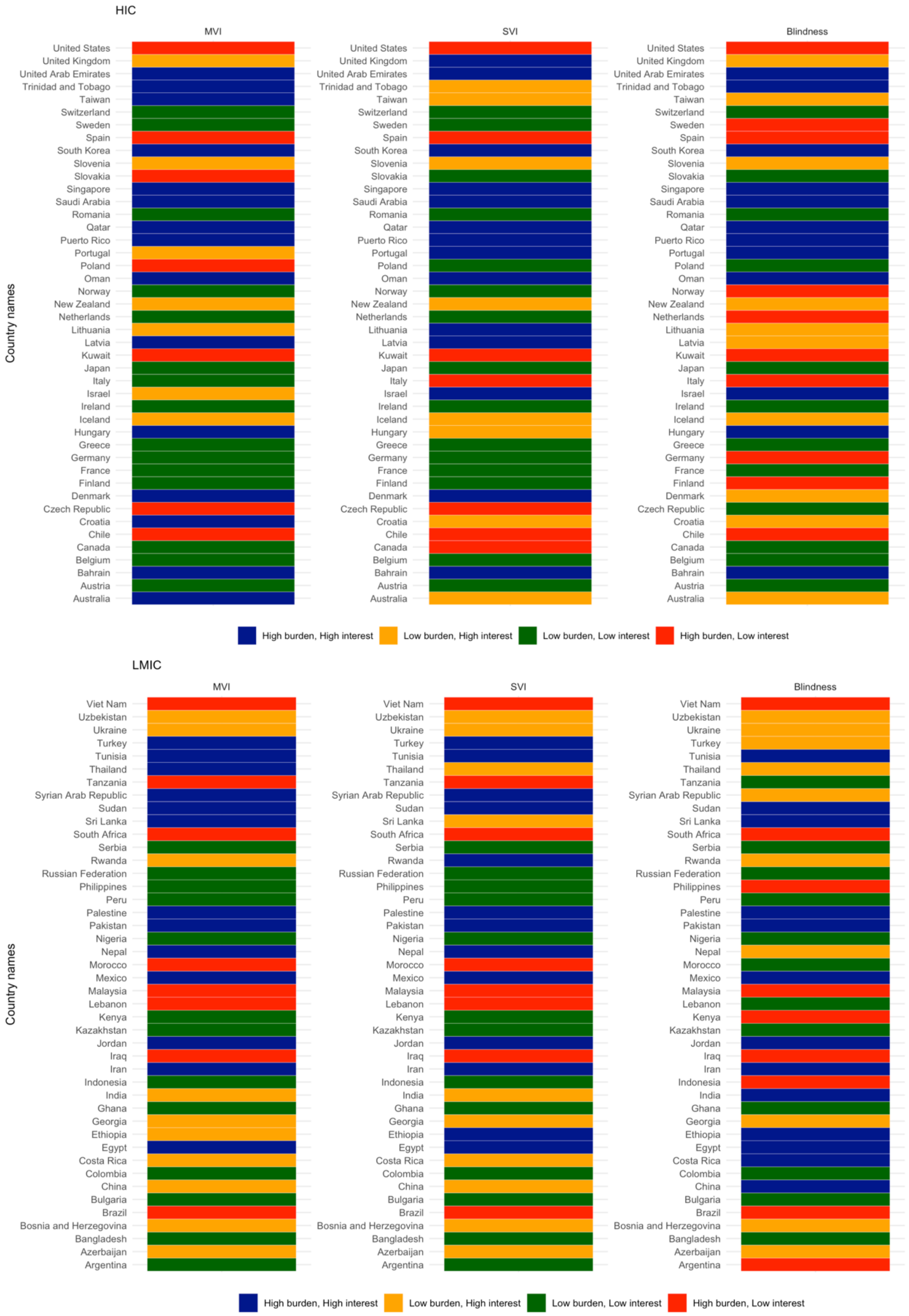

Supplement: Supplementary file 3 — contains Figures S1-S4. [file ijhpm-15-9345-s003.pdf]
